# Modeling the Impact of Uganda’s Safe Male Circumcision Program: Implications for Age and Regional Targeting

**DOI:** 10.1371/journal.pone.0158693

**Published:** 2016-07-13

**Authors:** Katharine Kripke, Andrea Vazzano, William Kirungi, Joshua Musinguzi, Alex Opio, Rhobbinah Ssempebwa, Susan Nakawunde, Sheila Kyobutungi, Juliet N. Akao, Fred Magala, George Mwidu, Delivette Castor, Emmanuel Njeuhmeli

**Affiliations:** 1 Health Policy Project, Avenir Health, Washington, D.C., United States of America; 2 Health Policy Project, Futures Group, Washington, D.C., United States of America; 3 Ministry of Health, Kampala, Uganda; 4 U.S. Agency for International Development (USAID), Kampala, Uganda; 5 U.S. Department of Defense, Kampala, Uganda; 6 Makerere University Walter Reed Project, Kampala, Uganda; 7 USAID, Washington, D.C., United States of America; Cardiff University, UNITED KINGDOM

## Abstract

**Background:**

Uganda aims to provide safe male circumcision (SMC) to 80% of men ages 15–49 by 2016. To date, only 2 million men have received SMC of the 4.2 million men required. In response to age and regional trends in SMC uptake, the country sought to re-examine its targets with respect to age and subnational region, to assess the program’s progress, and to refine the implementation approach.

**Methods and Findings:**

The Decision Makers’ Program Planning Tool, Version 2.0 (DMPPT 2.0), was used in conjunction with incidence projections from the Spectrum/AIDS Impact Module (AIM) to conduct this analysis. Population, births, deaths, and HIV incidence and prevalence were used to populate the model. Baseline male circumcision prevalence was derived from the 2011 AIDS Indicator Survey. Uganda can achieve the most immediate impact on HIV incidence by circumcising men ages 20–34. This group will also require the fewest circumcisions for each HIV infection averted. Focusing on men ages 10–19 will offer the greatest impact over a 15-year period, while focusing on men ages 15–34 offers the most cost-effective strategy over the same period. A regional analysis showed little variation in cost-effectiveness of scaling up SMC across eight regions. Scale-up is cost-saving in all regions. There is geographic variability in program progress, highlighting two regions with low baseline rates of circumcision where additional efforts will be needed.

**Conclusion:**

Focusing SMC efforts on specific age groups and regions may help to accelerate Uganda’s SMC program progress. Policy makers in Uganda have already used model outputs in planning efforts, proposing males ages 10–34 as a priority group for SMC in the 2014 application to the Global Fund’s new funding model. As scale-up continues, the country should also consider a greater effort to expand SMC in regions with low MC prevalence.

## Introduction

Three randomized trials have demonstrated that safe male circumcision (SMC, the term used by the Ministry of Health in Uganda to denote voluntary medical male circumcision) reduces the risk of HIV acquisition in men by approximately 60% [1–3], making it an essential element of a comprehensive HIV prevention program in settings with high rates of heterosexual HIV transmission and low prevalence of male circumcision (MC) [4]. Scaling up SMC to high levels of coverage offers substantial individual- and population-level benefits, including protective effects against HIV infection both for men and women and consequent reduced spending on care and treatment of HIV-positive individuals in the long term [4–6]. It has been estimated that expansion of SMC services to 80% coverage in high-priority settings could prevent up to 3.4 million new HIV infections over a period of 15 years [4].

With HIV prevalence estimated at 7.3% among the general population [7], Uganda is one of 14 priority countries identified for high-level SMC scale-up by the World Health Organization (WHO) and the Joint United Nations Programme on HIV/AIDS (UNAIDS) [8]. National estimates from 2011 indicate that 26% of men ages 15–49 are circumcised, with the largest proportions of circumcised men reporting having been circumcised during infancy (24.8%) or between the ages of 15 and 19 (23.9%) [7]. There is considerable geographic variation in MC prevalence, ranging from 2% in the Mid Northern region to 53% in the Mid Eastern region [7]. The proportion of men circumcised also differs significantly by religious status (97% of Muslim men vs. 10% of Catholic men), ethnicity (2% of Langi and Acholi men vs. 81% of Bagisu/Sabiny men), and economic status (35% of men living in wealthy households vs. 17% of men in poor households).

Uganda announced a Safe Male Circumcision Policy in 2010. In accordance with a recommendation from the World Health Organization (WHO) and the Joint United Nations Programme on HIV/AIDS (UNAIDS) for high-priority countries such as Uganda to reach and achieve 80% SMC coverage among 15- to 49-year-old males, the policy set a target of circumcising 4.2 million men ages 15–49 by 2016 [9, 10]. The country has made considerable progress toward this goal, with more than 2 million men reported to have been medically circumcised through December 2014, according to national indicators [4]. Estimates of the number of HIV infections averted by circumcisions conducted through 2014 by the national SMC program (including males ages 10–14) show that even if the country halted the SMC program, the circumcisions performed through 2014 would prevent an estimated 45,000 HIV infections by 2025. This estimate was derived through an analysis in another manuscript in this collection [11], which assumed that Uganda would scale up antiretroviral therapy (ART) coverage according to the 90-90-90 HIV treatment goals [12] during this period. (The 90-90-90 goals, advanced by UNAIDS, are an ambitious plan to control the HIV epidemic worldwide. By 2020, they call for 90 percent of people living with HIV to be diagnosed, 90 percent of those diagnosed to be on ART, and 90 percent of those on ART to be virally suppressed.)

Despite the achievements of Uganda’s SMC rollout, the country does not expect to meet its target by 2016, and additional efforts are required to attain 80% SMC coverage among 15- to 49-year-old males. Implementers have noted important trends in MC uptake that may have implications for program reach and success. Key among these are the large number of pre-adolescents ages 10–14 receiving SMC services in Uganda, a trend also noted in other priority countries in sub-Saharan Africa [13]. Uganda’s current circumcision target is based in part on modeled estimates of program impact from circumcising males ages 15–49 [4]. Clients ages 10–14 were not included when the national target was set, nor are they a focus of SMC outreach efforts. At the same time, a recent data quality assurance (DQA) exercise conducted by the Uganda Ministry of Health and the U.S. President’s Emergency Plan for AIDS Relief (PEPFAR) revealed that the number of clients over the age of 25 receiving SMC services is far lower than would be expected based on population estimates, suggesting unique barriers to demand creation for this subpopulation [14]. Program experiences such as these have led to interest in reconsidering Uganda’s age-specific SMC targets to better account for age disparities in the use of VMMC services.

Geographic variations both in HIV and MC prevalence in Uganda have also emerged as an important consideration in SMC programming, and in the HIV community as a whole [15–17]. The 2011 Uganda AIDS Indicator Survey demonstrates differences in HIV prevalence across the nine regions and between urban (10.7%) and rural (7.7%) areas of the country, and regional MC prevalence is uneven [7]. A 2010 situational analysis conducted in Kampala, Gulu, Kumi, and Rukungiri districts revealed wide variation in knowledge, attitudes, and opinions about MC among study participants, pointing to the need for varied, location-specific strategies to promote MC uptake [18]. The extreme variations in HIV and MC prevalence, use of services, and diverse attitudes about MC could have major implications for regional progress toward the national target.

Within this context, the study team undertook a modeling exercise to (a) update the estimated cost and impact of scaling up SMC among different age groups, including 10- to 14-year-olds, and (b) determine regional differences in cost-effectiveness and progress of scale-up. Results from these analyses will inform national SMC programming and strategic planning, by identifying age groups and/or geographic areas that require greater attention or investment. They will also help the country reassess its targets, based on progress to date by region and age group.

## Methods

### DMPPT 2.0 Model

The Decision Makers’ Program Planning Tool (DMPPT) 2.0 model is described in detail in another manuscript in this collection [19]. Briefly, DMPPT 2.0 is a simple, compartmental model implemented in Microsoft Excel 2010 that is designed to analyze the effects of age at circumcision on program impact and cost. The DMPPT 2.0 model tracks the number of circumcised males in newborns and in each five-year age group over time, taking into account age progression and mortality. The model calculates discounted SMC program costs and HIV infections averted in the population in each year in a user-specified SMC scale-up strategy, compared with a baseline scenario in which MC prevalence remains the same. The baseline scenario assumes that traditional or other circumcisions that produced the baseline MC prevalence continue at the same rate as before the SMC program was initiated.

#### Uganda data sources

All model inputs can be found in the supplemental materials (S1 and S2 Appendices). The DMPPT 2.0 model is populated with population, mortality, and HIV incidence and prevalence projections from an external source. For the Uganda country application, we used the national Spectrum/AIM model [20], which projects population size, mortality, and HIV prevalence and incidence based on data empirically collected from the country. The Uganda Ministry of Health provided the most recent validated version of the model, dated May 17, 2014. HIV prevalence data from surveillance sites used in Spectrum/AIM to project HIV incidence and prevalence were categorized by the eight out of the ten regions corresponding to those used in the Uganda 2011 AIDS Indicator Survey [21]. (Surveillance data were not available for the Central 1 region, and data from the Mid Eastern and East Central regions were combined in order to provide more surveillance sites to strengthen the projection.) HIV prevalence curves were fit to the surveillance data within each region using the R-spline fitting method [22] in the EPP3 module within Spectrum/AIM [20]. Population by age and year, mortality by age and year, annual number of male births, and HIV incidence by age and year were exported from this Spectrum/AIM file into a national Uganda DMPPT 2.0 file. For the regional analyses, a separate DMPPT 2.0 file was created for each region. The population-by-age projections for each year from the national file were multiplied by the ratio of the population in that region to the total national population. HIV incidence by age from the national file was multiplied by the ratio of the overall HIV incidence in that region (exported from the Spectrum/AIM file) to the national HIV incidence for each year between 2013 and 2020. For the years between 2021–2050, the regional to national incidence ratio for 2020 was used to scale the national incidence by age.

Numbers of SMCs conducted in the country in each region in each year were extracted from the national health management information system on April 2, 2014. The MC prevalence by age group in the model base year (2014) was derived from the Uganda Demographic and Health Survey 2011 [23]. The unit cost of SMC used in the analysis was $80 USD (all subsequent references to currency are in U.S. dollars), based on an expenditure analysis conducted by the PEPFAR team in Uganda. The annual per-person cost of antiretroviral therapy (ART) was $497, based on ART costs collected for an investment case analysis conducted in 2013. This cost reflects a weighted average of first- and second-line drugs, and also includes laboratory tests, staff, facilities, and above-facility-level costs for administration, logistics, training, planning, etc.

#### Analytical approaches

To examine the effect of client age on the impact of scaling up SMC, we created a series of scenarios in which each scenario had a target of 80% MC prevalence for a single age group or combination of age groups, leaving the target for the other age groups at the same level as the baseline. We created one scenario for each individual five-year age group, in addition to several scenarios with 80% targets for combined age groups, such as 10- to 34- or 15- to 29-year-olds. In each scenario, MC coverage was scaled up between 2014 and 2018, by applying a linear interpolation to the baseline MC prevalence for each age group in 2013 and the target coverage in 2018. After 2018, the coverage for each age group was maintained at the target level. For each scenario, we compiled the decrease in HIV incidence in the scale-up scenario compared with the baseline scenario in each year of the model, and the total number of circumcisions required during the scale-up phase (2014–2018). The following model outputs for each scenario were measured over the 15-year period between 2014 and 2028, inclusive: the total number of HIV infections averted in the population (including secondary HIV infections averted among females; see above); the number of SMCs per HIV infection averted; the total cost of the SMC program; the total number of HIV infections averted; and the total cost of the SMC program. Costs, numbers of circumcisions, and infections averted were all discounted at a rate of 3% per year. Uncertainty around the age distribution of HIV incidence was calculated as described in another manuscript in this collection [19].

To examine the differences in impact and cost-effectiveness across the different regions of Uganda, we compiled the cost per HIV infection averted over the 15-year period 2014–2018, inclusive, from each regional DMMPT 2.0 file. The SMC scale-up scenario used for this analysis was to scale up to 80% MC coverage among males ages 10–34. Uncertainty around the regional estimates was calculated as follows: The Spectrum/AIM Uncertainty Analysis tool [24] was run on the national Spectrum/AIM file described above. The median, lower 2.5%, and upper 97.5% bounds around the adult (ages 15–49 years old) HIV incidence for the year 2020 were extracted from the uncertainty tool. DMPPT 2.0 files for each region were created representing the lower and upper bounds of the HIV incidence, by multiplying the incidence in the original file by the ratio of the lower to median or upper to median values. Cost per HIV infection averted extracted from the lower bound file for each region was used as the lower bound in the analysis, and likewise for the higher bound.

## Results

### Age Analyses

The effects of client age on the SMC program were measured according to four different metrics: (1) the number of SMCs required to avert one HIV infection over a period of 15 years (SMC/ HIV infections averted [IA]); (2) the immediacy of impact (decrease in HIV incidence over five years); (3) the magnitude of impact (number of HIV infections averted over 15 years); and (4) the cost-effectiveness, measured as cost per HIV infection averted over a period of 15 years. The period of 15 years (2014–2028, inclusive) is long enough to discern appreciable impact in HIV incidence and short enough to be relevant to policymakers.

**Fig 1:**
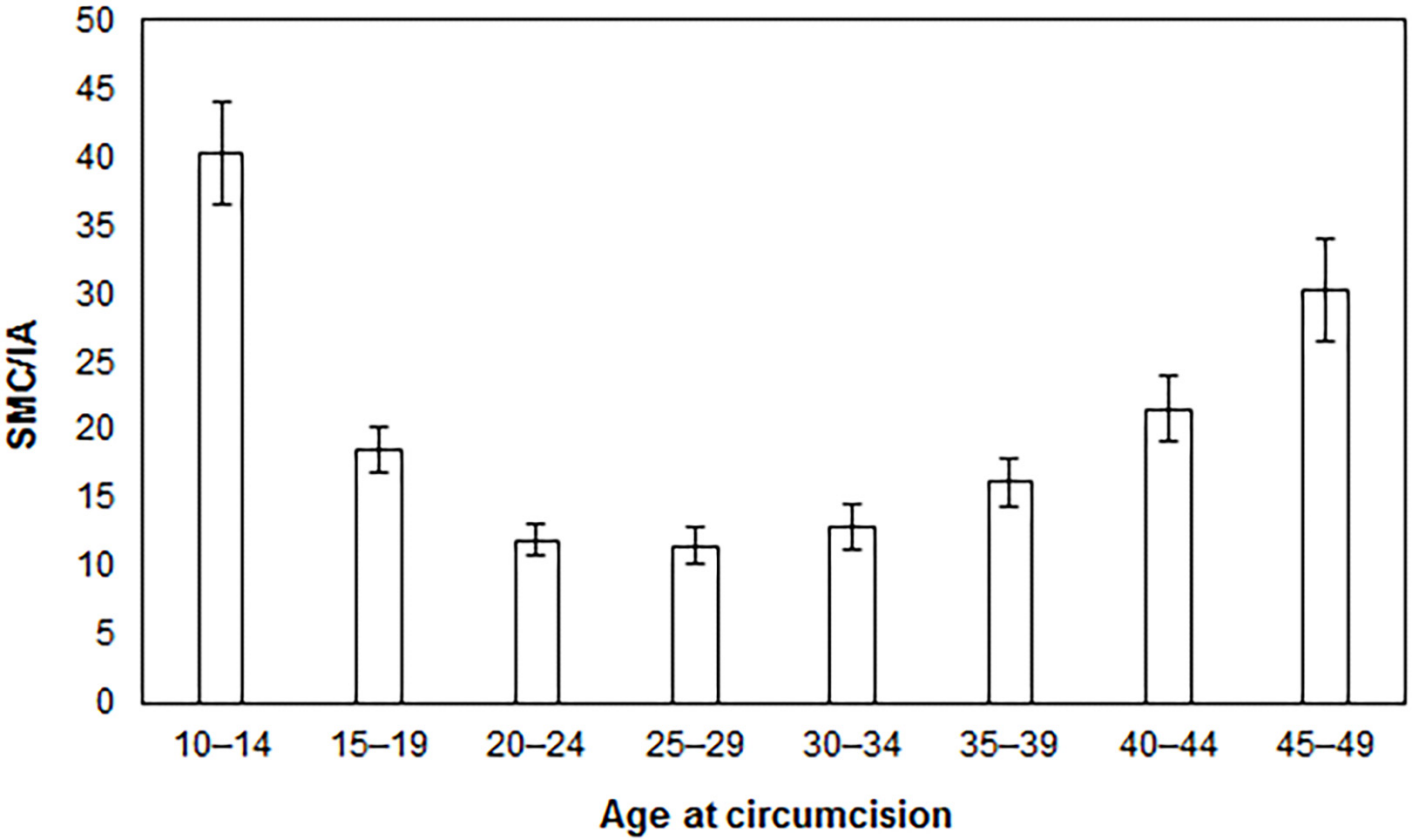
Safe medical circumcisions required per HIV infection averted (SMC/IA) by age-specific scale-up strategy, 2014–2028.

Fig 1 indicates the number of SMCs required to prevent one HIV infection in Uganda from 2014 to 2028, by five-year age groups. For each age group depicted on the x-axis, the model assumes a scenario in which circumcisions are performed for men in that age group only; however, the number of HIV infections averted is projected out over the entire population, because the model follows men as they age over time. Although the primary impact of SMC is on the number of new HIV infections in men, HIV infection in women is also affected through a reduced probability that a woman will come into contact with an HIV-positive man [4], an effect the model takes into account. The fewest SMCs per HIV infection averted (IA) for the period from 2014 to 2028 occur in the 20- to 24- (12 SMC/IA), 25- to 29- (12 SMC/IA), and 30- to 34-year-olds (13 SMC/IA) age groups.

**Fig 2:**
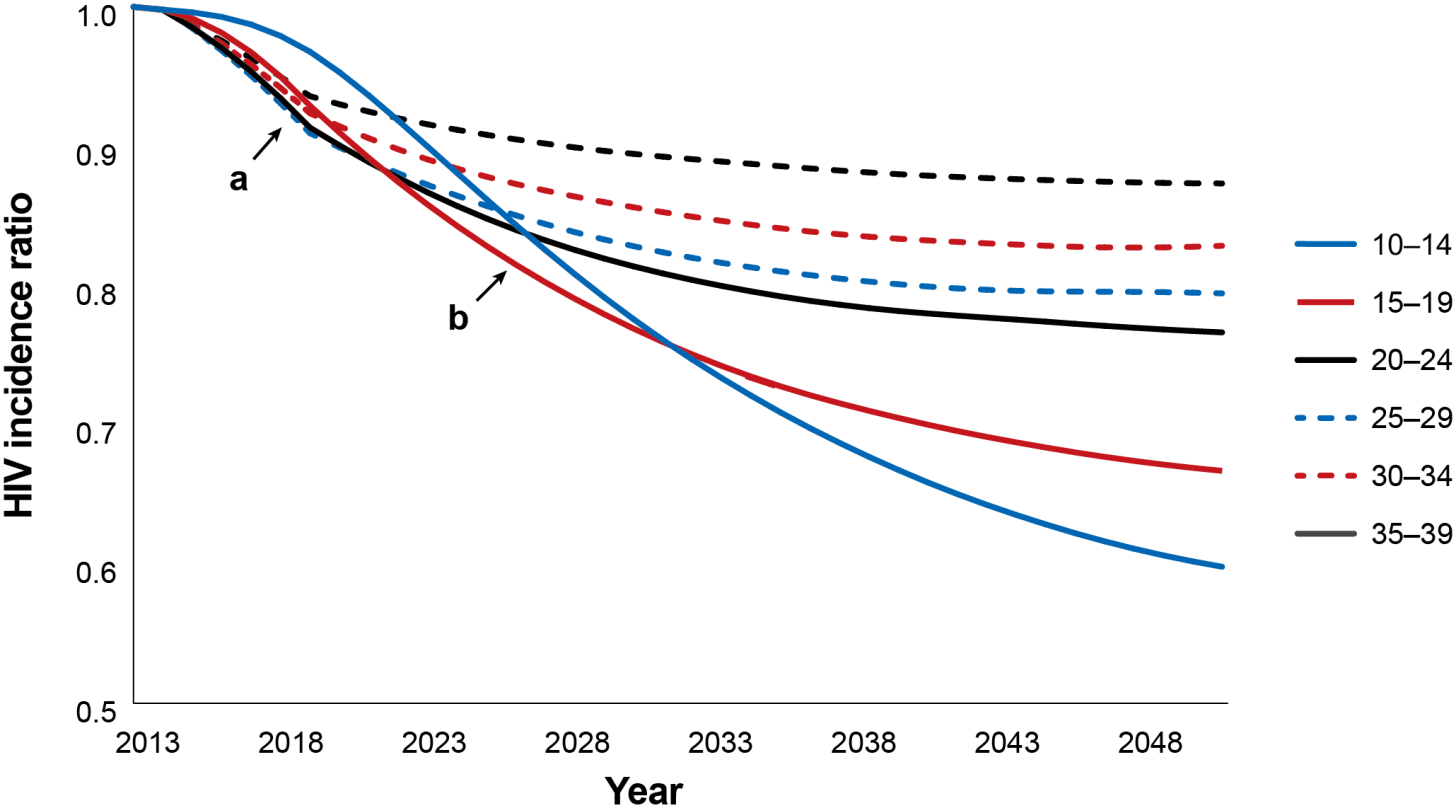
Immediacy and magnitude of impact: incidence rate reductions by age-specific scale-up strategy, 2014–2048. (a) immediacy of impact (5 years). (b) magnitude of impact (15 years).

The impact of SMC programs on HIV incidence rates in the short-term (i.e., immediacy of impact) is presented in Fig 2 (marker a). Similarly to the analysis on SMC/IA, each line corresponds to a circumcision strategy in which only that age group is circumcised, with the impact followed over time in the entire population. The reduction in new HIV infections under each age-specific scale-up strategy relative to the reduction expected with no scale-up strategy appears on the y-axis. Over a five-year period after 2013, the most rapid reductions in incidence can be seen in the strategies scaling up SMC among 20- to 24-, 25- to 29-, and 30- to 34-year-olds.

Next we considered the magnitude of impact after 15 years (2014–2028, inclusive). When considering individual five-year age groups, the greatest reduction in HIV incidence after 15 years occurs among 10- to 14- and 15- to 19-year-olds (Fig 2, marker b).

**Fig 3:**
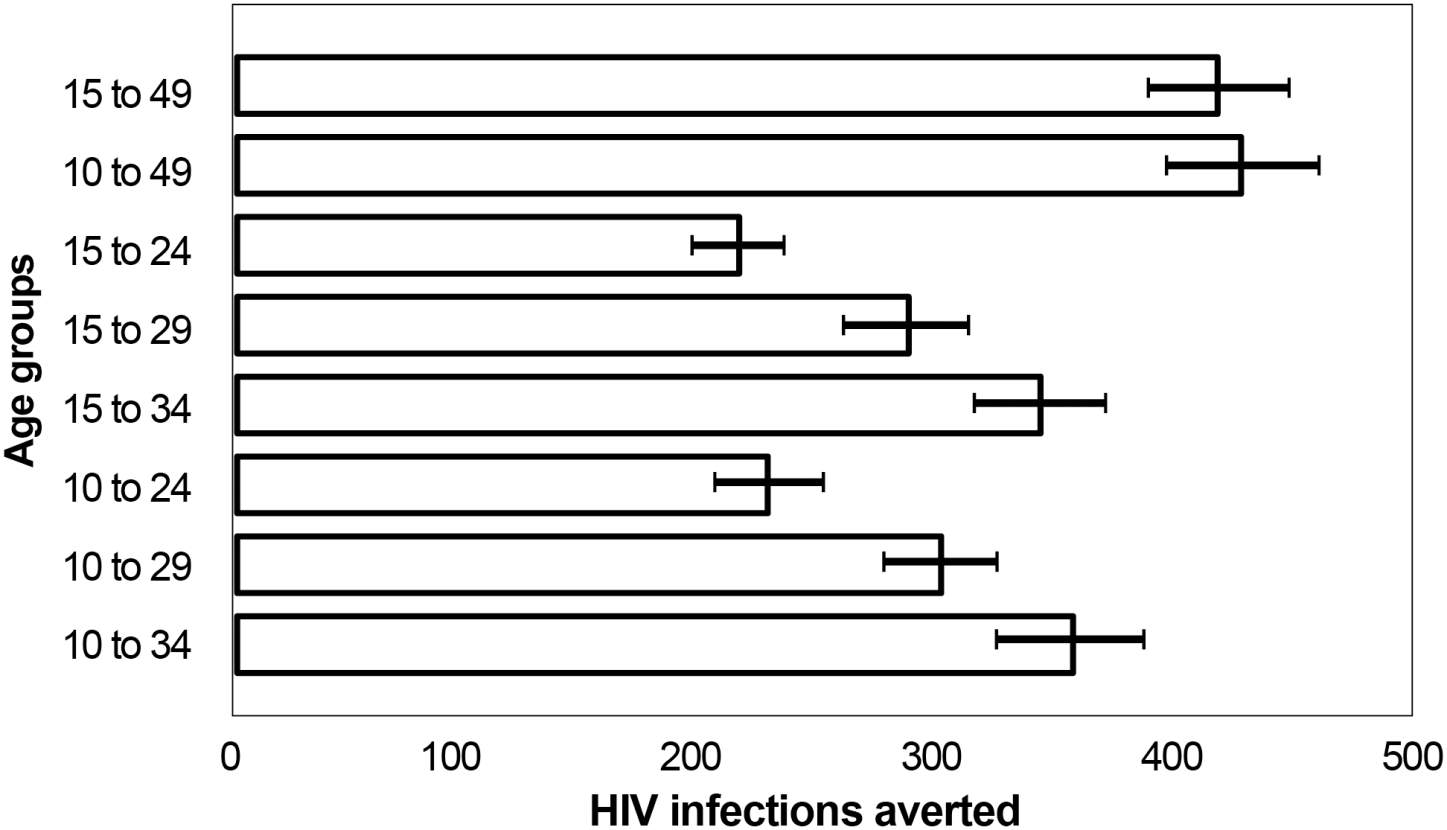
Magnitude of impact: total HIV infections averted by age-specific scale-up strategy, 2014–2028.

Though the previous analyses examined fictitious scenarios in which only a single five-year age group was circumcised, we aimed to assess the impact and cost-effectiveness of circumcising age groups that might be closer to actual implementation strategies. We therefore examined several scenarios with 80% targets for combined age groups, such as 10–34 or 15–29. Fig 3 shows the magnitude of impact of different age-targeting strategies, defined as the total number of HIV infections averted over 15 years. The topmost bar depicts the current strategy in Uganda in which MC coverage is scaled up to 80% among all men ages 15–49. This would result in averting 479,558 new HIV infections. If a narrower age group is needed for programmatic reasons, scaling up SMC among clients ages 10–34 will still result in 85% (402,000) of HIV infections averted, compared with scaling up VMMC among clients ages 15–49. The narrower the age group, the fewer circumcisions that are conducted and the lower the magnitude of impact. Thus scale-up among the 15- to 24- and 10- to 24-year-olds would have the least impact over 15 years, averting only 242,000 and 257,000 HIV infections, respectively. To achieve the greatest magnitude of impact, the country would have to scale up to 80% coverage among the entire 10- to 49-year-old male population (485,000 HIV infections averted). In all cases, strategies with age 10 as the lower age bound result in slightly larger numbers of HIV infections averted compared with strategies with a lower bound of age 15, but the differences between these two groups are less than the uncertainty around the estimates.

**Fig 4:**
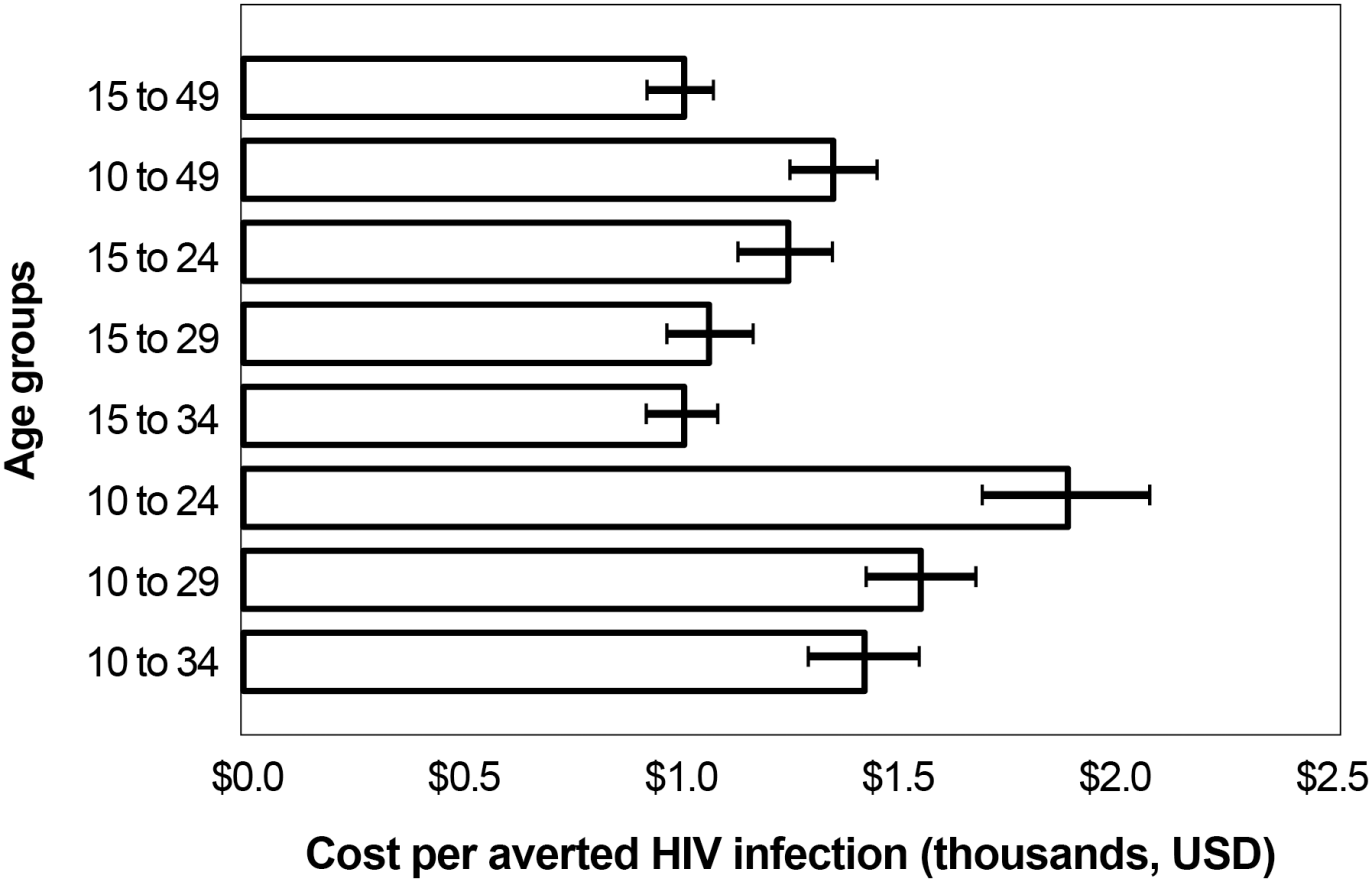
Cost-effectiveness: safe male circumcision cost per HIV infection averted by age-specific scale-up strategy, 2014–2028.

To determine cost-effectiveness, SMC cost per HIV infection averted between 2014 and 2028 for each age-targeted strategy was examined alongside that for the country’s current strategy (15- to 49-year-olds, represented in the topmost bar in Fig 4). The most cost-efficient strategy is circumcising men ages 15–34, which is projected to cost the same per HIV infection averted as the baseline scenario ($1,100). The least efficient strategy in this figure is focusing on 10- to 24- year-olds, which would cost approximately $2,100. The cost per HIV infection averted is uniformly higher in the age groups starting with 10-year-olds, because boys ages 10–14 are mostly not at risk until they become sexually active a few years later, so they are not reaping the protective benefits of circumcision during part of the 15-year period measured.

Table 1 summarizes the best age group to target according to each metric, the number of circumcisions needed to scale up to the 80% target between 2014 and 2018, and the estimated total cost between 2014 and 2028 for each age-targeting strategy.

**Table 1:** Summary of Priority Age Groups for Safe Medical Circumcision Scale-up for Each Metric in DMPPT 2.

Parameter	Priority Age Group	# Circumcisions Needed by 2018	Cost
			(Unit Cost of $80 USD)
SMC/IA	20–34 yrs	2,160,553	$291,415,904
Immediacy of Impact	20–34 yrs	2,160,553	$291,415,904
Magnitude of Impact	10–19 yrs	3,227,418	$494,570,541
Cost-Effectiveness	15–34 yrs	3,311,676	$428,188,349
Country Age-Targeting Strategy 2010–2016	15–49 yrs	4,565,828	$522,595,419

### Geographic analyses

**Fig 5:**
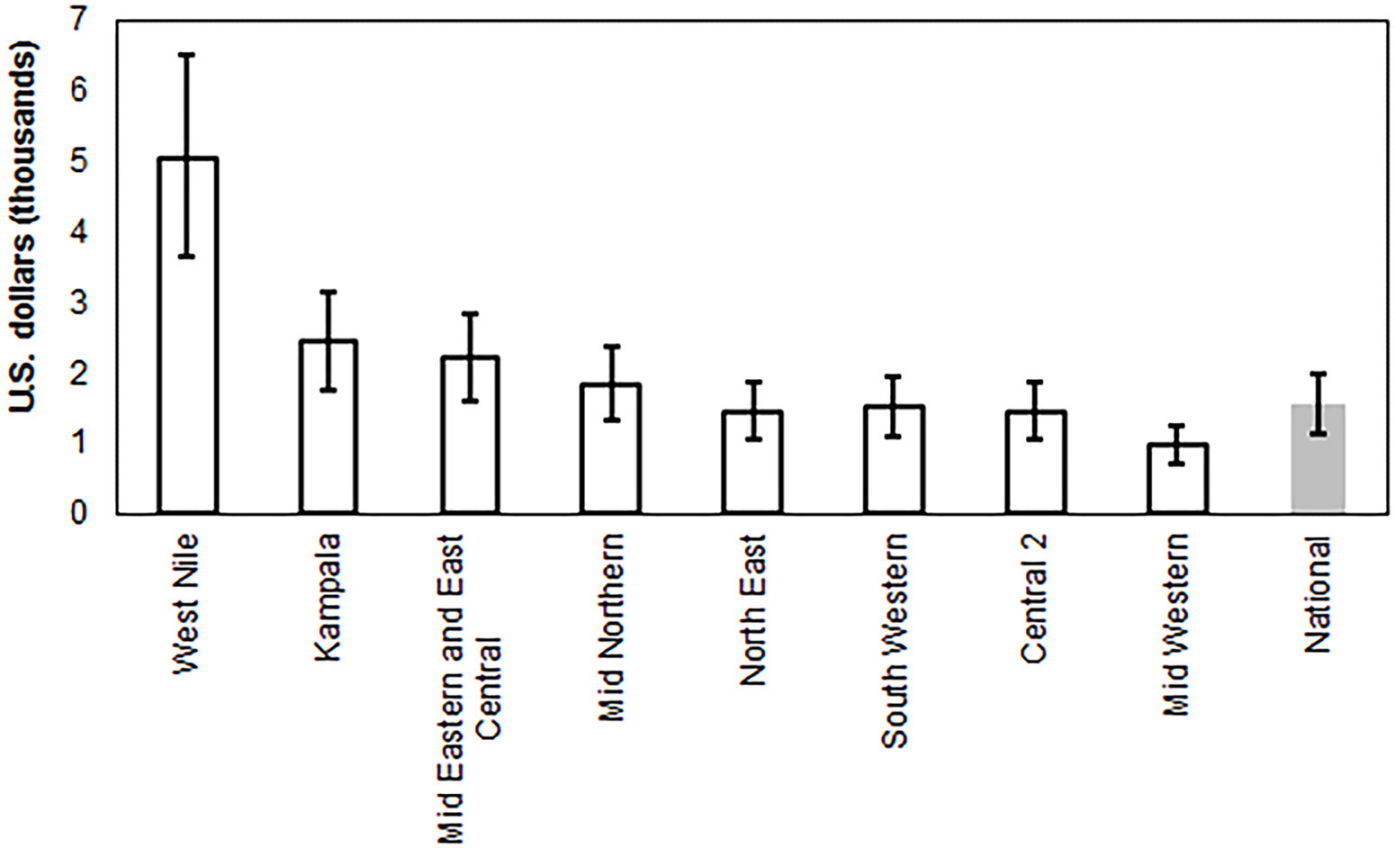
Discounted cost per HIV infection averted among 10- to 34-year-olds by region, 2013–2028.

Regional DMPPT 2.0 projections were created in order to look at variations in cost-effectiveness and progress toward the SMC target nationally and across Uganda’s nine regions. Though the current national target includes men ages 15–49, this analysis examined scale-up among men ages 10–34 in order to inform the country’s Global Fund application. The SMC cost per HIV infection averted between 2014 and 2028 for each region is presented in Fig 5. Uncertainty bounds for each region overlap those of the national estimate for all regions except West Nile, suggesting that variations in cost-effectiveness across most regions are not certain. An analysis of treatment costs averted by scaling up SMC among males ages 10–34 over the same period demonstrates that the strategy is cost-saving across all regions including West Nile (e.g., the future costs of care and treatment in a scenario without scale-up are greater than the cost of SMC scale-up in 10- to 34-year-olds) (S1 Fig). This finding still holds when the VMMC unit cost is $160 instead of $80 and the annual ART cost remains the same.

**Fig 6:**
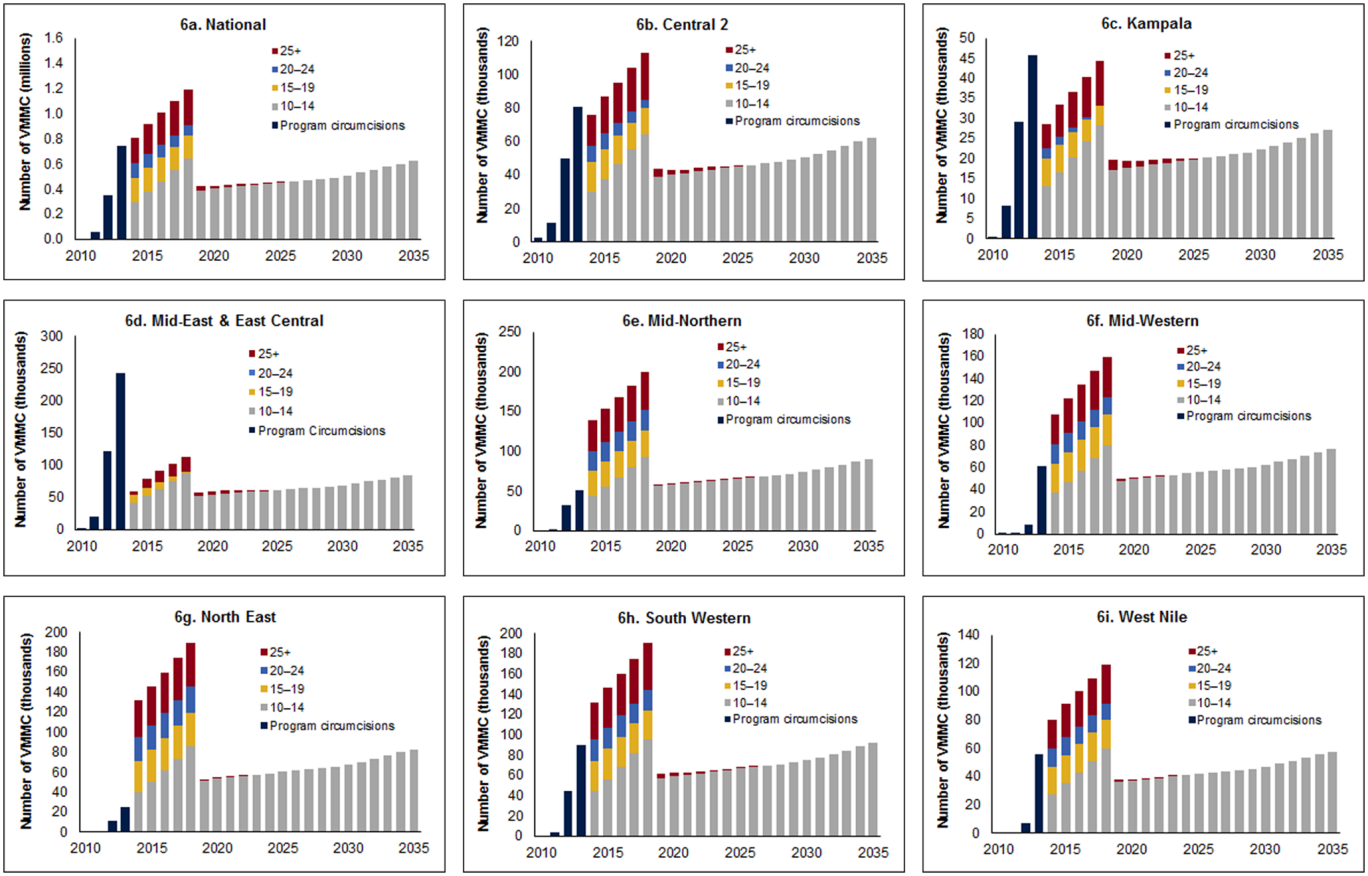
Number of VMMCs required, by age group and year. 6a. National. 6b. Central 2 region. 6c. Kampala. 6d. Mid East and East Central. 6e. Mid Northern. 6f. Mid Western. 6g. North East. 6h. South Western. 6i. West Nile.

Fig 6 displays the SMC program implementation progress to date nationally and for each region. Years prior to 2014 show the number of circumcisions conducted by the region’s SMC program each year, with age groups differentiated by color. The subsequent bars display the number of circumcisions that would need to be conducted in each age group for each year through 2018 in order to successfully reach and maintain 80% coverage among men ages 10–34. Overall, regions varied in their rate of progress. Uganda National, Central 2, Kampala, Mid East, and East Central regions are ahead of reaching the projected targets, while Mid Western, South Western, and West Nile appear to be on target, assuming scale-up continues at the same pace. Two regions (Mid Northern and North East) lag behind the others and may not achieve target coverage unless the SMC program is increased substantially in these regions.

## Discussion

Results from this modeling exercise reaffirm that while the country has made progress toward its goal of reaching 80% SMC coverage for men ages 15–49 years old, this target will be difficult to achieve by 2016, if at all, given the challenges with reaching men over age 35. In examining the impact of scaling up SMC among different age groups and the current progress of scale-up at the regional level, the DMPPT 2.0 points to areas where more strategic allocation of resources may accelerate progress. In particular, the exercise reveals that even if Uganda attains 80% coverage of this age group at a national level, a substantial number of SMCs will be required annually (approximately 450,000 per year) to maintain this coverage level, suggesting a need for planning to sustain the VMMC program over the long term.

In the age-targeted strategies, modeled estimates for each of the four metrics (SMC/IA, immediacy of impact, magnitude of impact, and cost-effectiveness) resulted in different, but largely overlapping, recommended priority age groups, ranging from ages 10 to 34. The model shows that circumcising 20- to 34-year-olds will have the fastest impact on HIV incidence and will require the fewest circumcisions for each HIV infection averted, which is compatible with evidence from other studies in sub-Saharan Africa showing that men are at highest risk for HIV infection when they are in their twenties and thirties [25].

Circumcising men ages 15–34 represents the best age-targeting strategy in terms of cost-effectiveness, with the SMC cost per HIV infection averted coming to $1,100. This is the same SMC/IA cost if the program were to circumcise 80% of men in the larger 15- to 49-year age range. While scaling up SMC across the entire 10- to 49-year age range will achieve the greatest number of HIV infections averted over 15 years (485,000, magnitude of impact), this wide age range presents a number of logistic and resource constraints to the SMC program, and does not provide guidance on priority subgroups. Additionally, demand for SMC among older men (>35 years) is lower than among younger men, and achieving 80% coverage among 36- to 49-year-olds is unlikely [14]. Therefore, should the SMC program be unable to reach clients older than 35, it could still prevent 402,000 HIV infections over 15 years by focusing on clients ages 10 to 34 (that is, 83% of the impact achieved by scaling up to 80% SMC coverage among 10- to 49-year-olds).

Stakeholders in Uganda were interested in understanding how the inclusion of 10- to 14-year-olds in the SMC program target would impact program cost and effectiveness, particularly in light of the need for accelerated progress toward targets. Circumcising males ages 10–14 does not provide an immediate impact on HIV incidence in the population, as the average age of sexual initiation, and therefore HIV exposure, is 18.4 years for men [7], and peak HIV incidence is between the ages of 25 and 39 (S2 Fig). Circumcising males ages 10–14 also increases the program’s resource requirements. Nevertheless, inclusion of males ages 10–14 will provide the greatest long-term impact on HIV incidence and the most HIV infections averted. As adolescents have a right to safe, high-quality healthcare, and the country has seen that males ages 10–14 are seeking SMC services, even without demand creation for this group, inclusion of this age group in country SMC targets may be necessary to better reflect the actual client base.

It will be essential for decision makers to consider model results alongside the priorities of Uganda’s SMC program as well as country context and trends. For example, uptake of circumcision among men over the age of 25 is notably lower than projections of eligible clients would indicate [14], yet the DMPPT 2.0 suggests that reaching clients ages 25–34 will increase the overall impact and cost-effectiveness of the program. Program implementers may therefore wish to invest in strategies to increase uptake among this age group.

West Nile is the only region where the SMC program appeared less cost-effective than in other regions and nationally, because incidence projections there were lower. Estimated cost per HIV infection averted is a function of the number of SMCs per HIV infection averted: the more procedures required to avert one HIV infection, the higher the cost. Furthermore, the number of SMCs required to prevent one HIV infection is inversely related to incidence, as more procedures are required to avert an HIV infection in areas where incidence is low. It is possible that estimates of incidence for West Nile region are unreliable; the Spectrum curve shows discrepancy in HIV prevalence trends between household surveys and sentinel surveillance sites (S3 Fig). In any case, the model predicted that scaling up SMC in West Nile is still cost-saving, so it would be beneficial to continue the SMC program there even if it is true that the program in West Nile is relatively less cost-effective.

Finally, we demonstrated that the progress of the SMC program has varied across regions, with Mid Northern and North East regions lagging behind all others. Both regions have low baseline MC prevalence compared to other regions and the country at large, with only 1.6% of men circumcised in Mid Northern region and 6.7% in North East region, compared to 10% to 53% in other regions. Accelerated efforts are needed to reach 80% coverage of MC among those ages 10–34 in these areas, and the country may need to consider allocating additional resources there.

### Outcomes and policy implications

The DMPPT 2.0 is a strategic planning tool, meant to support—though not dictate—SMC programming and priorities. This analytic exercise was conducted in partnership with Uganda’s Ministry of Health, and has resulted in various tangible outcomes. The revised targets arising from the DMPPT 2.0 analysis for men who need to be circumcised in order to attain 80% coverage at the national level were used in the country’s recent application to the Global Fund to Fight AIDS, Tuberculosis and Malaria’s new funding model, which proposed males ages 10–34 as a priority age group for the SMC program. Notably, no specific strategies will be employed to promote uptake among men ages 35 and above, though MC services will remain available to these clients.

The exercise also brought to light issues related to collection and reporting of programmatic data. At the time of the DMPPT 2.0 analysis, the research team found that five-year age distributions of SMC program circumcisions were not available in any of the collated reporting data. Most sites in Uganda use a paper-based tally for documentation, and age information was reported in aggregate into the national health information system for ages 0–<2, 2–<5, 5–<15, 15–49, and >49. Starting in April 2015, the country began using a new health management information system that collects SMC data for ages <1, 1–9, 10–14, 15–19, 20–24, 25–49, and 50-plus. This new system will enable the collection of more granular SMC client age data, which can be used to more closely monitor and manage the program.

Finally, a DQA exercise conducted in 2014 by PEPFAR and the Ministry of Health uncovered wide variation in the age distributions of circumcisions in different clinics. It would be useful to confirm and investigate the reasons for these variations, so that implementers can learn what programmatic variables result in attracting different ages of clients, and therefore tailor the program to reach males in the age group targets determined by policy makers.

## Limitations

This study has a number of limitations. The model was designed for ease of use within countries, and therefore relies on available national and regional estimates of demographic and epidemiological data. As such, modeled projections are subject to the biases and assumptions of these inputs (e.g., baseline MC prevalence, unit costs, and projections of future HIV incidence). In particular, as future HIV incidence is dependent on many biological and behavioral influences, it is not possible to accurately project long-term HIV incidence.

An additional limitation exists for the SMC unit cost used in the model. Cost assumptions are based on a fixed unit cost of $80 and therefore do not reflect possible differences in cost related to client age, geographic location, implementation model, phase of scale-up, or other factors (e.g., demand-creation activities, waste management, etc.). For example, the SMC unit cost may be higher for older men, who require more demand-creation activities, and one might obtain different results in terms of the most cost-effective ages. Total cost estimates for scale-up scenarios are therefore useful only for relative comparisons, and should not be used for budget projections.

The model does not account for broader social, cultural, and logistical barriers to program acceptability and implementation. When selecting a scale-up strategy or target, Uganda will need to consider the model in the context of other influences, such as human resources, the political environment, and challenges to demand creation specific to certain geographic areas or subpopulations.

## Conclusions

The SMC program in Uganda has made appreciable progress toward the goal of achieving 80% MC coverage among males ages 15–49, though the country will not reach this target by 2016. Our DMPPT 2.0 exercise shows that focusing SMC efforts on specific age groups and/or regions may help to accelerate and ensure continued progress. According to the model, the country may want to consider changing its target to focus on males ages 10–34 (while still providing SMC to anyone medically eligible, regardless of age) and putting greater effort to expand SMC in regions with low baseline MC prevalence. Policymakers in Uganda have already used DMPPT 2 outputs in planning efforts, proposing males ages 10–34 as a priority group for the SMC program in the country’s 2014 application to the Global Fund’s new funding model. As scale-up continues, Uganda should continue to take into account model outputs alongside the broader country context to develop high-impact SMC programming.

## Supporting Information

S1 Appendix Uganda DMPPT 2.0 Model Inputs, Part 1. See Methods section for data sources.
(DOCX)

S2 Appendix Uganda DMPPT 2.0 Model Inputs, Part 2 (epidemiology and demographic inputs). See Methods section for data sources
(XLSX)

S1 Fig Comparison of safe male circumcision (SMC) program costs and HIV treatment costs averted scaling up SMC among 10- to 34-year-olds, by region (2015 to 2028). (TIF)

S2 Fig Age-specific HIV incidence for males in Uganda, 2013. (TIF)

S3 Fig Spectrum curve-fitting for West Nile to estimate HIV prevalence. Green triangles represent HIV prevalence at sentinel surveillance sites. Red diamonds with error bars represent HIV prevalence estimates and confidence bounds from population-based surveys; these are used to adjust the curves generated from the sentinel surveillance data. Gray lines represent HIV prevalence curves generated by the fitting algorithm, with the best-fitting curve indicated by the red line with “+” markers and the uncertainty bounds of the curves represented by the dotted blue lines.
(TIF)

## Abbreviations

ART: antiretroviral therapy
DMPPT: Decision Makers’ Program Planning Tool
DQA: data quality assurance
IA: HIV infections averted
MC: male circumcision
PEPFAR: U.S. President’s Emergency Plan for AIDS Relief
PPD ARO: Partners in Population and Development, Africa Regional Office
PRB: Population Reference Bureau
SMC: safe male circumcision
UNAIDS: Joint United Nations Programme on HIV/AIDS
USAID: United States Agency for International Development
USD: U.S. dollars
VMMC: voluntary medical male circumcision
WHO: World Health Organization
WRA: White Ribbon Alliance
