# Correction: Modeling the Impact of Uganda's Safe Male Circumcision Program: Implications for Age and Regional Targeting

**DOI:** 10.1371/journal.pone.0169699

**Published:** 2017-01-03

**Authors:** Katharine Kripke, Andrea Vazzano, William Kirungi, Joshua Musinguzi, Alex Opio, Rhobbinah Ssempebwa, Susan Nakawunde, Sheila Kyobutungi, Juliet N. Akao, Fred Magala, George Mwidu, Delivette Castor, Emmanuel Njeuhmeli

There is an error in the Funding statement. The number for the Cooperative Agreement Project SOAR (Supporting Operational AIDS Research) is incorrect. The correct number is OAA-A-14-00060.
