# Supplementary material for: Modeling the Impact of Uganda’s Safe Male Circumcision Program: Implications for Age and Regional Targeting
Source: PLoS One. 2016 Jul 13;11(7):e0158693. doi: 10.1371/journal.pone.0158693 (PMC4943628; doi:10.1371/journal.pone.0158693)
Supplement: S1 Appendix — See Methods section for data sources. (DOCX) [file pone.0158693.s001.docx]

| Input | Data | Reference |
| --- | --- | --- |
| Percent circumcised in base year, by 5-year age group | EIMC: 16.7%  5-9: 20.6%  10-14: 23.2%  15-19: 22.3%  20-24: 29.3%  25-29: 28.3%  30-34: 25.5%  35-39: 25.5%  40-44: 22.2%  45-49: 22.2%  50-54: 22.2%  55-59: 22.2% | Uganda Demographic and Health  167 Survey 2011 (age 15-49)  Authors’ assumptions (EIMC, 5-9, 10-14, 50-54, 55-59) |
| Number of male circumcisions Performed, 2010-2013 | 2010: 9,052  2011: 57,132  2012: 352,039  2013: 742,978 | National program statistics |
| VMMC effectiveness | 0.6 | Auvert B, Taljaard D, Lagarde E, Sobngwi-Tambekou J, Sitta R, et al. (2005) Randomized, controlled intervention trial of male circumcision for reduction of HIV infection risk: The ANRS 1265 trial. PLoS Med 2(11): e298.  Gray RH, Kigozi G, Serwadda D, Makumbi F, Watya S, Nalugoda N et al. Male circumcision for HIV prevention n men in Rakai, Uganda: a randomized trial. Lancet 2007; 369: 767-66.  Bailey RC, Moses S, Parker CB, Agot K, Maclean I, Krieger JN, et al. Male circumcision for HIV prevention in young men in Kisumu, Kenya: a randomized controlled trial. Lancet 2007; 369: 643-56. |
| Ratio of infections averted among females to males | 0.53 | Spectrum, Goals Model |
| HIV incidence | See S2 Appendix – Spectrum Inputs | Authors’ Calculations  Spectrum, AIDS Impact Module and Goals module |
| Discount rate | 0.03 | Authors’ assumption. |
