# Supplementary figures and images for: Modeling the Impact of Uganda’s Safe Male Circumcision Program: Implications for Age and Regional Targeting
Source: PLoS One. 2016 Jul 13;11(7):e0158693. doi: 10.1371/journal.pone.0158693 (PMC4943628; doi:10.1371/journal.pone.0158693)

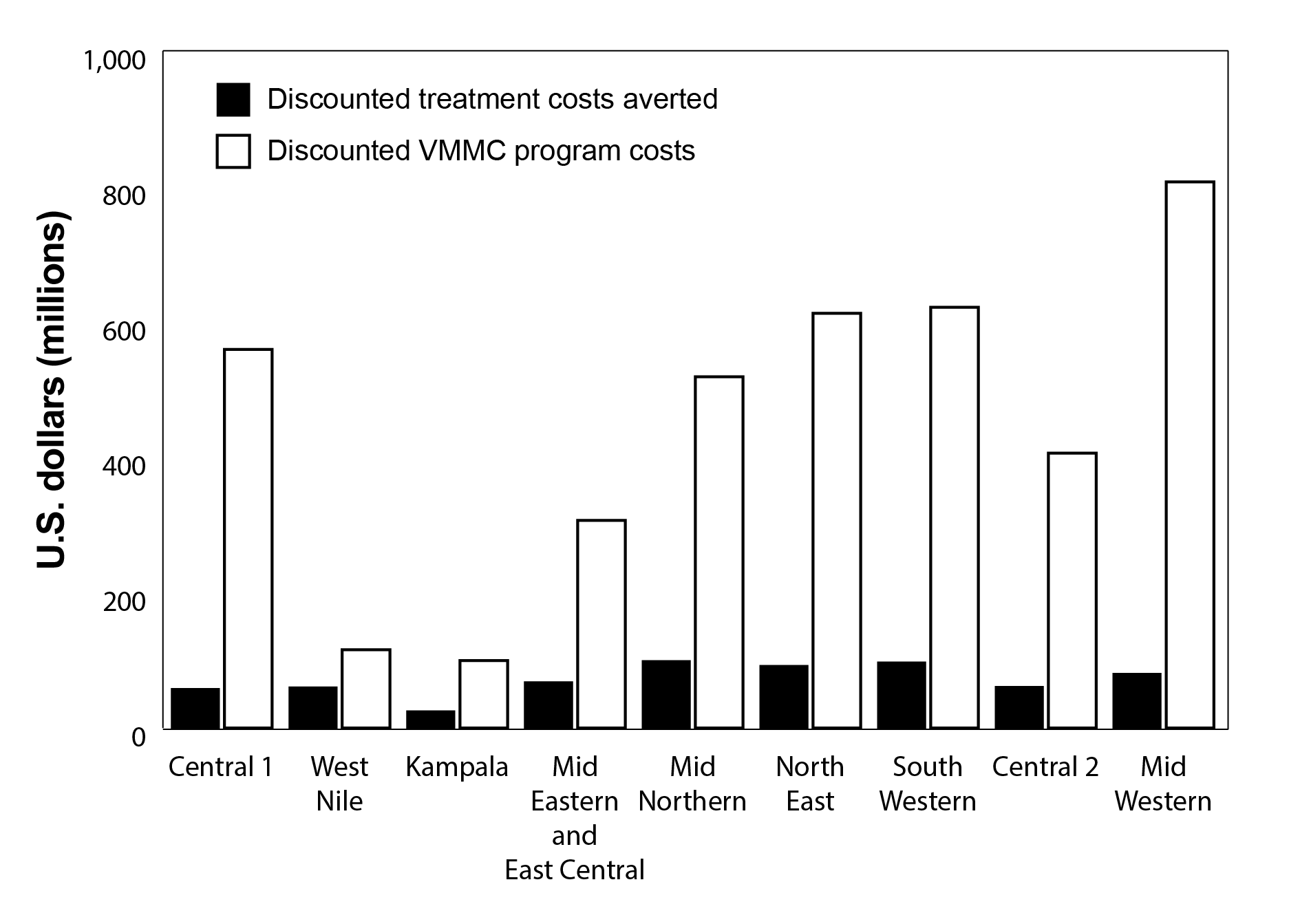

Supplement: S1 Fig — (TIF) [file pone.0158693.s003.tif]

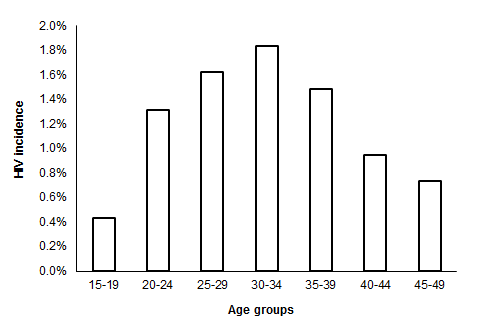

Supplement: S2 Fig — (TIF) [file pone.0158693.s004.tif]

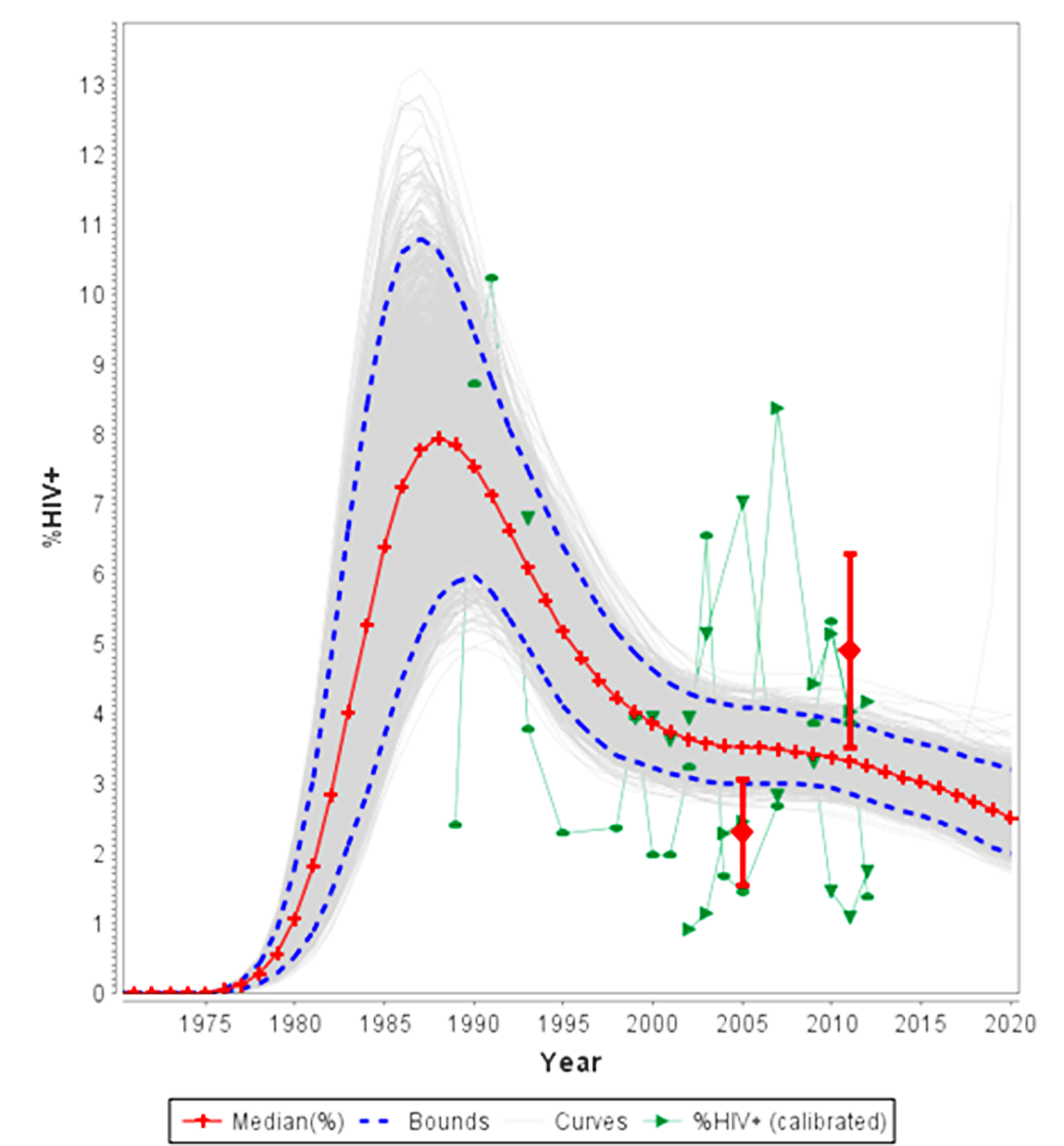

Supplement: S3 Fig — Green triangles represent HIV prevalence at sentinel surveillance sites. Red diamonds with error bars represent HIV prevalence estimates and confidence bounds from population-based surveys; these are used to adjust the curves generated from the sentinel surveillance data. Gray lines represent HIV prevalence curves generated by the fitting algorithm, with the best-fitting curve indicated by the red line with “+” markers and the uncertainty bounds of the curves represented by the dotted blue lines. (TIF) [file pone.0158693.s005.tif]
